# Analysis of Mycosporine-Like Amino Acids in Selected Algae and Cyanobacteria by Hydrophilic Interaction Liquid Chromatography and a Novel MAA from the Red Alga *Catenella repens*

**DOI:** 10.3390/md13106291

**Published:** 2015-10-09

**Authors:** Anja Hartmann, Kathrin Becker, Ulf Karsten, Daniel Remias, Markus Ganzera

**Affiliations:** 1 Institute of Pharmacy, Pharmacognosy, University of Innsbruck, Innrain 80-82, Innsbruck 6020, Austria; E-Mail: Anja.Hartmann@uibk.ac.at; 2 Division of Biological Chemistry, Biocenter, Medical University of Innsbruck, Innrain 80-82, Innsbruck 6020, Austria; E-Mail: Kathrin.Becker@i-med.ac.at; 3 Institute of Biological Sciences, Applied Ecology & Phycology, University of Rostock, Albert-Einstein-Str. 3, Rostock 18059, Germany; E-Mail: Ulf.Karsten@uni-rostock.de; 4 University of Applied Sciences Upper Austria, Stelzhammerstr. 23, Wels 4600, Austria; E-Mail: Daniel.Remias@fh-wels.at

**Keywords:** mycosporine-like amino acids, HILIC, LC-MS, validation, quantification, isolation, UV-sunscreen

## Abstract

Mycosporine-like amino acids (MAAs), a group of small secondary metabolites found in algae, cyanobacteria, lichens and fungi, have become ecologically and pharmacologically relevant because of their pronounced UV-absorbing and photo-protective potential. Their analytical characterization is generally achieved by reversed phase HPLC and the compounds are often quantified based on molar extinction coefficients. As an alternative approach, in our study a fully validated hydrophilic interaction liquid chromatography (HILIC) method is presented. It enables the precise quantification of several analytes with adequate retention times in a single run, and can be coupled directly to MS. Excellent linear correlation coefficients (*R*^2^ > 0.9991) were obtained, with limit of detection (LOD) values ranging from 0.16 to 0.43 µg/mL. Furthermore, the assay was found to be accurate (recovery rates from 89.8% to 104.1%) and precise (intra-day precision: 5.6%, inter-day precision ≤6.6%). Several algae were assayed for their content of known MAAs like porphyra-334, shinorine, and palythine. Liquid chromatography-mass spectrometry (LC-MS) data indicated a novel compound in some of them, which could be isolated from the marine species *Catenella repens* and structurally elucidated by nuclear magnetic resonance spectroscopy (NMR) as (*E*)-3-hydroxy-2-((5-hydroxy-5-(hydroxymethyl)-2-methoxy-3-((2-sulfoethyl)amino)cyclohex-2-en-1-ylidene)amino) propanoic acid, a novel MAA called catenelline.

## 1. Introduction

Solar radiation reaching the Earth’s surface affects numerous biological functions in living organisms, and extensive exposure to UV-B (280–315 nm) and UV-A (315–400 nm) can cause significant stress and deleterious effects at the cellular level. Adaptation mechanisms are therefore required for any living organism, and they have been studied extensively for higher plants already [1,2]. However, micro and macro algae and cyanobacteria follow different strategies and several unique constituents for UV protection are known in this respect [3,4,5,6]. Mycosporine-like amino acids (MAAs), a group of small water-soluble compounds are especially relevant. Their photo protective potential can be explained by extremely high molar extinction coefficients up to 50,000, and to date more than 20 MAAs with absorption maxima between 309 and 360 nm have been identified [7,8,9]. Just recently, due to more refined isolation and analytical techniques, novel or partially characterized MAAs were reported [10,11]. For the HPLC separation of MAAs reversed phase materials have been utilized to date [12,13]. However, satisfactory separations, especially of highly polar derivatives, required either different methods for individual MAAs [10,14] or the use of two reversed-phase columns in tandem [11]. Alternatively, our study describes a totally new approach for MAA analysis by HILIC (hydrophilic interaction liquid chromatography). This technique is described as a variant of normal phase chromatography, and separations are dependent on a compound’s polarity and its degree of solvation. HILIC is particularly useful for the analysis of highly polar compounds and it is an interesting alternative to the previously reported methods for MAA analysis. Moreover, to the best of our knowledge none of the established procedures have been validated, which is currently common practice in analytical sciences. The described method is also suitable for LC-MS studies, which proved helpful in assigning several known MAAs, but also to identify potentially new structures in diverse marine and terrestrial algae. One previously unknown derivative with a molecular mass of 382 was isolated from the red alga *Catenella repens* (*C. repens*) (Lightfoot) Batters, and it showed to be a novel MAA with an amino-cyclohexenimine structure. The marine alga *C. repens* has a wide biogeographic distribution in Europe, North and South America, and preferentially grows on sheltered shady rocks or soil between high tide levels and supralitoral zones.

## 2. Results

### 2.1. Method Development

**Figure 1:**
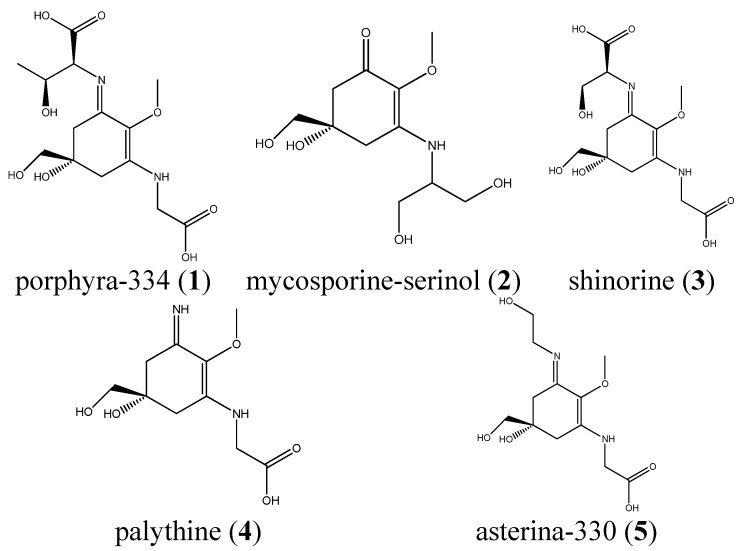
Chemical structures of the available MAA standards, which were isolated from *Palmaria palmata*, *Porphyra* sp., *Lichina pygmea* and *Plectropomus leopardus*.

**Figure 2:**
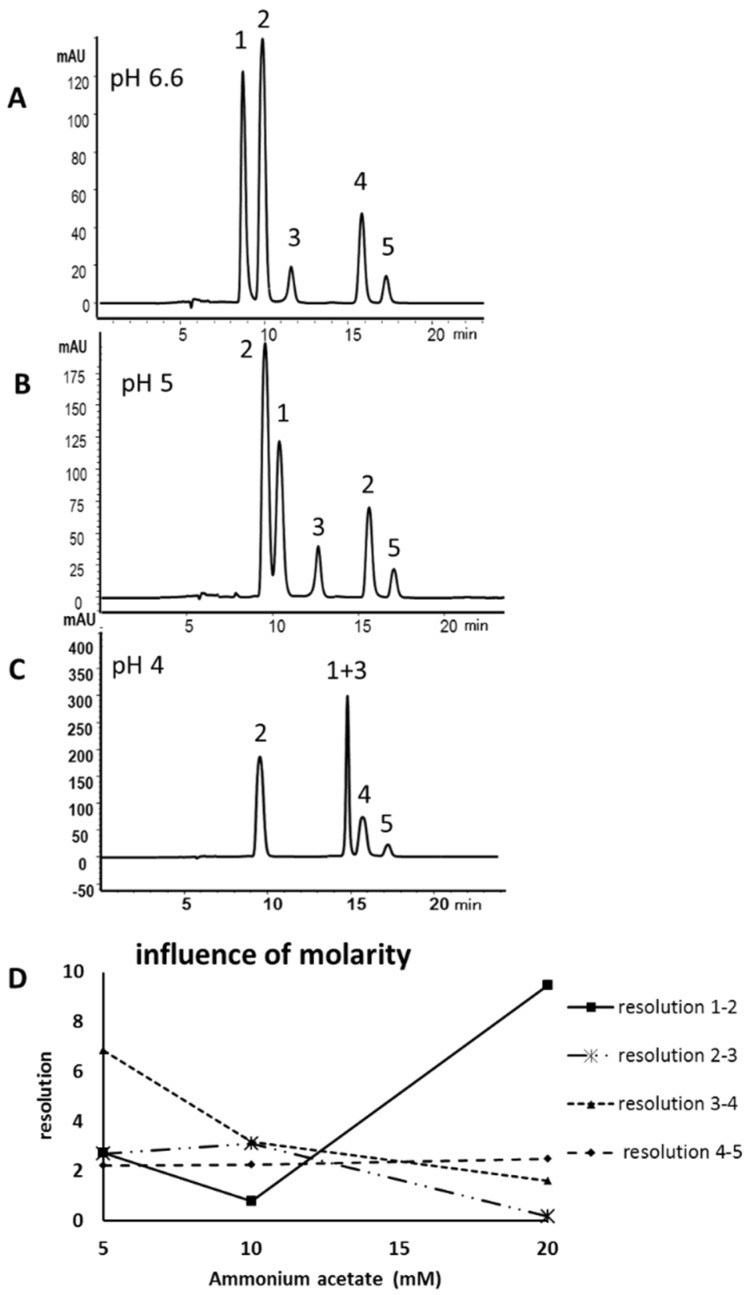
Hydrophilic interaction liquid chromatography (HILIC) separation of mycosporine-like amino acids (MAAs) **1** to **5** at the optimum pH value of 6.6 (**A**); or using a mobile phase with pH 5 (**B**); or pH 4 (**C**); and (**D**) the influence of buffer molarity (5 mM to 20 mM ammonium acetate) on the results. Peak assignment is according to Figure 1.

For the development of a HILIC method five MAAs (see Figure 1 for structures) were isolated as previously described [15] and used as standards. Three different stationary phases were available for an initial screening, a zwitterionic HILIC from Merck, Darmstadt, Germany (Sequant ZIC-HILIC), and two core-shell materials, a Kinetex HILIC (Phenomenex, Aschaffenburg, Germany) and a HILIC Poroshell 120 from Agilent, (Waldbronn, Germany). All columns had identical dimensions (150 mm × 4.6 mm) and the particle size was comparable (2.6–3.5 µm). The latter column yielded the best results concerning separation efficiency and peak shape, resulting in an optimum separation within less than 20 min (Figure 2A). Porphyra-334 (**1**) eluted first (8.9 min), followed by mycosporine-serinol (**2**; 10.0 min), shinorine (**3**; 11.9 min), palythine (**4**; 15.9 min) and asterina-330 (**5**; 17.2 min). Water/acetonitrile mixtures with ammonium acetate as an additive were well suited as mobile phases, but only when the buffer concentration and pH-value were carefully optimized. As can be seen in Figure 2B,C, at a pH other than the optimum of 6.6 (native pH) a decline in selectivity was observed. At pH 5.0 the resolution of **1** and **2** decreased, whereas at pH 4.0 **2** and **3** overlapped. Thus, in contrast to RP-HPLC the addition of acid is not advantageous in this application. Mycosporine-like amino acids are zwitterionic substances, which is relevant because HILIC phases are well known to exhibit ion-exchange mechanism too. Thus, because of their structure containing two amino acid residues, compounds **1** and **3** are strongly influenced by changes in pH, whereas the other MAAs remained relatively unaffected. The second most crucial factor was buffer molarity (Figure 2D). At a buffer concentration above 5 mM ammonium acetate in both mobile phases the resolution of most peak pairs, with the exception of compounds **1** and **2**, gradually decreased. At 20 mM, co-elution of **1** and **3** was observed. A less relevant factor was column temperature; its chromatographic influence is shown in the Supplementary Materials (Figure S1); 20 °C was selected as the overall best resolutions were obtained at this temperature.

### 2.2. Validation

The method was validated by establishing calibration curves of the three standards, **1**, **3** and **4**; for **2** and **5**, sufficient material was not available. Excellent linear correlation coefficients (*R*^2^ ≥ 0.9991) were obtained within a concentration range of 250–3.91 µg/mL for **1** and **4**, and from 125 to 1.95 µg/mL for **3**. All calibration data are summarized in Table 1. LOD (limit of detection) and LOQ (limit of quantitation) values were found to be between 0.16 and 0.43 μg/mL, and from 0.48 to 1.31 μg/mL, respectively. Selectivity of the method was assured by no visible co-elutions (shoulders) in the relevant signals, LC-MS (liquid chromatography–mass spectrometry) data, and by very consistent UV-spectra within the relevant peaks (as confirmed by the peak purity option in the operating software). Intra-day (RSD = 5.6%) and inter-day precision (RSD ≤ 6.6%) were within accepted limits. Accuracy was assured by spiking accurately weighted samples of *L. foveolarum* with three different concentrations of **1**, **3** and **4**. This alga did not contain MAAs and was therefore chosen as a blank matrix for respective experiments. For all compounds, the observed recovery rates were acceptable and ranged from 96.8% to 104.1%. However, for **3**, the recovery at the low spike range was approximately 10% lower than the theoretical value. This might be explained by integration inconsistencies at this low concentration.

**Table 1:** Validation of the HILIC method.

Calibration Data for Porbphyra, Shinorine and Palythine							
Substance	Regr. Equation		Corr. Coefficient	σ _rel_ of Slope	Range (µg/mL)	LOD ^1^ (µg/mL)	LOQ ^2^ (µg/mL)
1	*y* = 87.37*x* + 96.00		*R*^2^ = 0.9999	0.36	250–3.91	0.30	0.91
3	*y* = 71.72*x* + 28.95		*R*^2^ = 0.9991	0.59	125–1.96	0.16	0.48
4	*y* = 40.99*x* + 36.81		*R*^2^ = 0.9999	0.18	250–3.91	0.43	1.31
**Accuracy and Precision of the Assay**							
	**Accuracy ^3^**			**Precision ^4^**			
**Substance**	**High Spike**	**Medium Spike**	**Low Spike**	**Day 1**	**Day 2**	**Day 3**	**Intra-day**
1	104.14	104.13	102.75	/	/	/	
3	97.90	98.69	89.84	/	/	/	
4	102.36	96.77	101.02	44.79 (6.56)	44.16 (1.67)	49.95 (5.72)	46.30 (5.57)

^1^ LOD: limit of detection as determined with purified standards; ^2^ LOQ: limit of quantification as determined with purified standards; ^3^ expressed as recovery rates in percent (sample *Leptolyngbya foveolarum*); ^4^ values reflect µg/mL, relative standard deviations are given in brackets (*n* = 5, sample *Palmaria palmata*).

### 2.3. Isolation of Catenelline from Catenella repens

**Figure 3:**
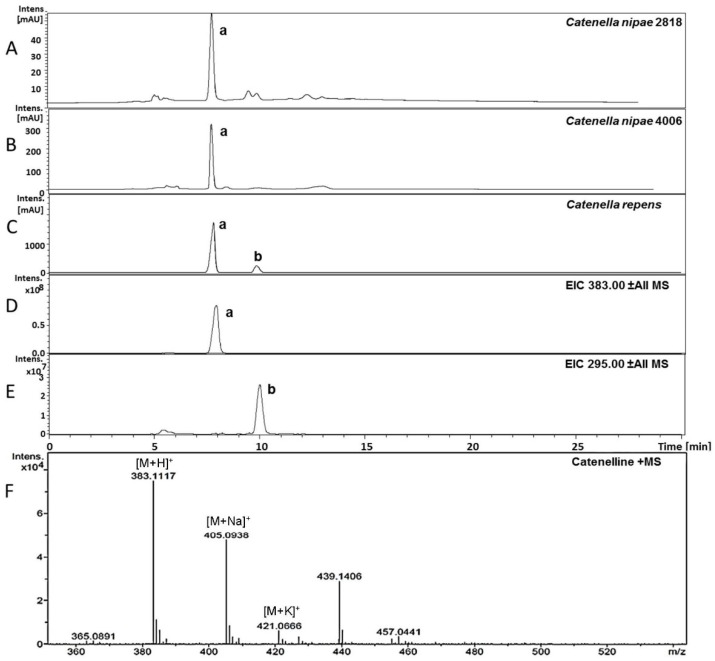
Identification of novel MAAs by LC-MS in *Catenella* *nipae* (4006 and 2818) and *Catenella repens*. Chromatograms (**A**) to (**C**) are recorded at 320 nm; traces **D** and **E** indicate *m*/*z* values of **a** (383) and **b** (295) in positive ESI mode. The high-resolution MS spectra of catenelline in positive ESI mode is shown as (**F**).

**Figure 4:**
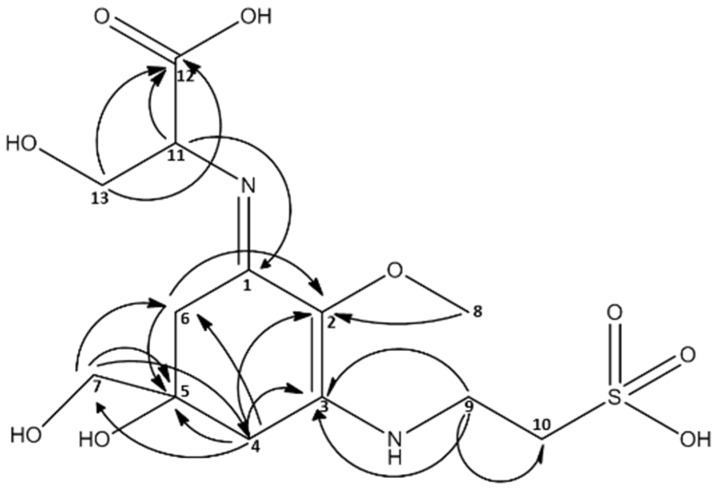
Structure of catenelline, a novel MAA isolated from *Catenella repens*. NMR long-range correlations are indicated with arrows.

The crude extracts of several species from the red algal genus *Catenella* (Order: *Gigartinales*) were investigated by HPLC-MS. The respective results indicated the presence of MAAs; not the known ones (**1**–**5**), but two single peaks with MAA typical UV-maxima and previously not reported molecular mass. Compound **a** showed an absorption maximum at 334 nm and a *m*/*z* value of 383 ([M + H]^+^; adducts with Na, K and water were visible too), and a minor constituent **b** at 320 nm and *m*/*z* = 295 ([M + H]^+^), respectively (Figure 3). The same observations were already reported by Karsten *et al.*, who tentatively assigned these compounds as MAAs [16]. Because sufficient biomass was available, *Catenella repens* was selected for the isolation of compound **a**. First, the crude extract was pre-purified on activated carbon cartridges, and then a final separation was possible by semi-preparative HPLC using a HILIC stationary phase. The purity of the so obtained compound was confirmed by LC-MS, and the structure elucidated by NMR (see full data set in Supporting Material, Figures S2–S6). Characteristic NMR shifts (Table 2) confirmed the presence of a MAA with an amino-cyclohexenimine scaffold, 2D-NMR experiments indicated two side chains attached to carbon atoms 1 (serine) and 3 (taurine). Their position was confirmed by long-range correlations visible in the HMBC spectra, and relevant connectivities are indicated by arrows in Figure 4. Both side chains have already been found individually in other MAAs, for example serine in shinorine and taurine in mycosporine tau. The latter was discovered by Stochaj *et al.*, in 1994 [12]. As the NMR data of all substructures were in good agreement to literature values, compound **a** was finally identified as (*E*)-3-hydroxy-2-((5-hydroxy-5-(hydroxymethyl)-2-methoxy-3-((2-sulfoethyl)amino)cyclohex-2-en-1-ylidene)amino) propanoic acid, a new MAA for which we propose the trivial name “catenelline” (high resolution ESI-MS data corresponding to [M + H]^+^ = 383.1117 (mass error 2 ppm), ε = 18800, MS/MS fragmentation pattern: Figure S7).

**Table 2:** NMR shift values for catenelline; spectra were recorded on a 600 MHz NMR instrument in deuterated water.

MAA *m*/*z* = 383 [M + H]^+^		
	^13^C	^1^H
1	162.03	-
2	128.46	-
3	162.78	-
4	35.53	2.98 d (17.4) ; 2.91 d (17.5)
5	73.74	-
6	36.16	2.93 d (17.4); 2.70 d (17.3)
7	70.31	3.60 d
8	62.14	3.66 s
9	42.02	3.88 m
10	52.54	3.27 dt (1.0, 6.5)
11	63.39	4.33 dd (3.8, 6.7)
12	177.37	-
13	65.40	3.98 m (6.4, 4.4)

The second possibly new MAA in this alga, compound **b**, was present in a much lower amount and its isolation is tedious and currently in progress. However, first NMR data indicated an MAA with an imino-cyclohexenone structure.

### 2.4. Quantitative Analysis of Samples

Several different samples (marine and terrestrial algae, cyanobacteria; Table 3) were available for quantification. Prior to analysis, dried material (approximately 0.2 g per sample) was extracted using the protocol described by Carreto *et al.* [11]; see Section 4.4 for details. After the last extraction step, the remaining material was extracted once more and the solution analyzed by HPLC, in order to ensure an exhaustive procedure. For quantification each sample was measured in triplicate (RSD always ≤3.4%), and the amount of MAAs was calculated using the calibration curves of the respective standards. For other MAAs, either known or tentatively identified compounds by LC-MS, the calibration data of **4** was used for quantification.

**Table 3:** Origin of analyzed samples.

Species	Origin of Sample
**Marine algae**	
*Porphyra* sp*.*	commercially available; Asia Express Food, LOT-Nr. 120516, Kampen, NL
*Porphyra* ssp*.*	commercially available; Porto Muinos, LOT-Nr. 2117543, Cambre, E
*Palmaria palmata*	commercially available; Irish Seaweeds, LOT-Nr. 5391513420184, Belfast, UK
*Lichina pygmea*	1998, Millers landing, Victoria, Australia, collected and identified by U.K., University of Rostock, Germany, provided in 2013
*Catenella repens*	2002, Roscoff, Brittany, collected and identified by U.K., University of Rostock, Germany, provided in 2013
*Catenella nipae* 2818	1987, Cowie Beach, Queensland, Australia, collected and identified by John West; since 2000 grown by U.K., University of Rostock, Germany and provided in 2013
*Catenella nipae* 4006	1999, Maningrida, Arnhem Land, Northern Territory, Australia, collected and identified by John West; since 2000 grown by U.K., University of Rostock, Germany and provided in 2013
*Catenella caespitosa* 2689	1984, La Parguera, Puerto Rico, collected and identified by John West; since 2000 grown by U.K., University of Rostock, Germany and provided in 2013
**Terrestrial algae**	
*Macrochloris multinucleata*	EPSAG Culture Collection of Algae, University of Göttingen, Germany; Strain-Nr. 39.96
**Cyanobacteria**	
*Nostoc commune*	Culture Collection of Autotrophic Organism, Třeboň, Czech Republic; isolated by Vinatzer 1975; strain-Nr. Innsbruck V157
*Calothrix* sp*.*	Culture Collection of Autotrophic Organisms, Třeboň, Czech Republic; isolated by Zehnder 1977; strain-Nr. 034; GenBank: L05609.1
*Leptolyngbya foveolarum*	Culture Collection of Autotrophic Organisms, Třeboň, Czech Republic; isolated by ZEHNDER 1965; strain-Nr. 081; GenBank: AM398970.1

**Figure 5:**
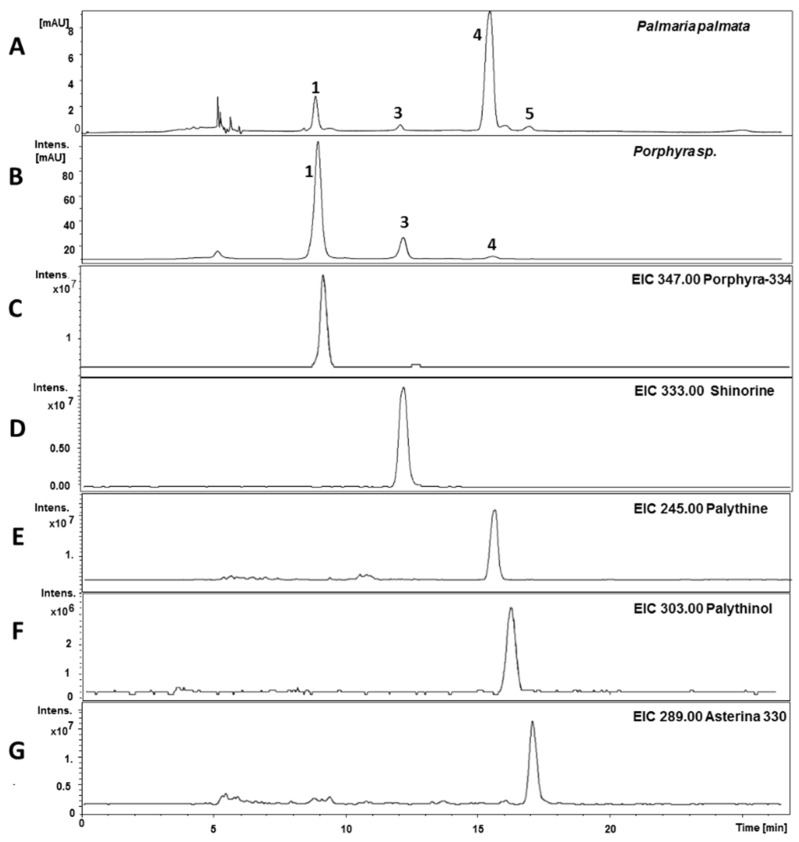
Determination of MAAs in *Porphyra* sp*.* and *Palmaria palmata*; chromatograms (**A**,**B**) were recorded at 320 nm; and the other traces (**C**–**G**) show the assignment of individual compounds in *P. palmata* by LC-MS in EIC mode.

**Figure 6:**
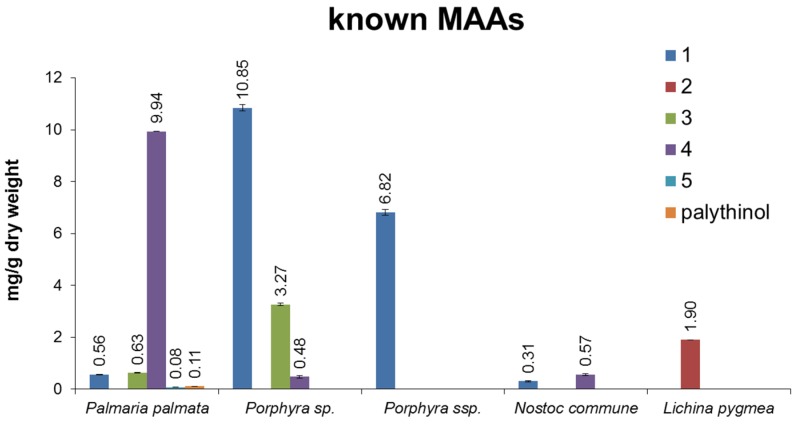

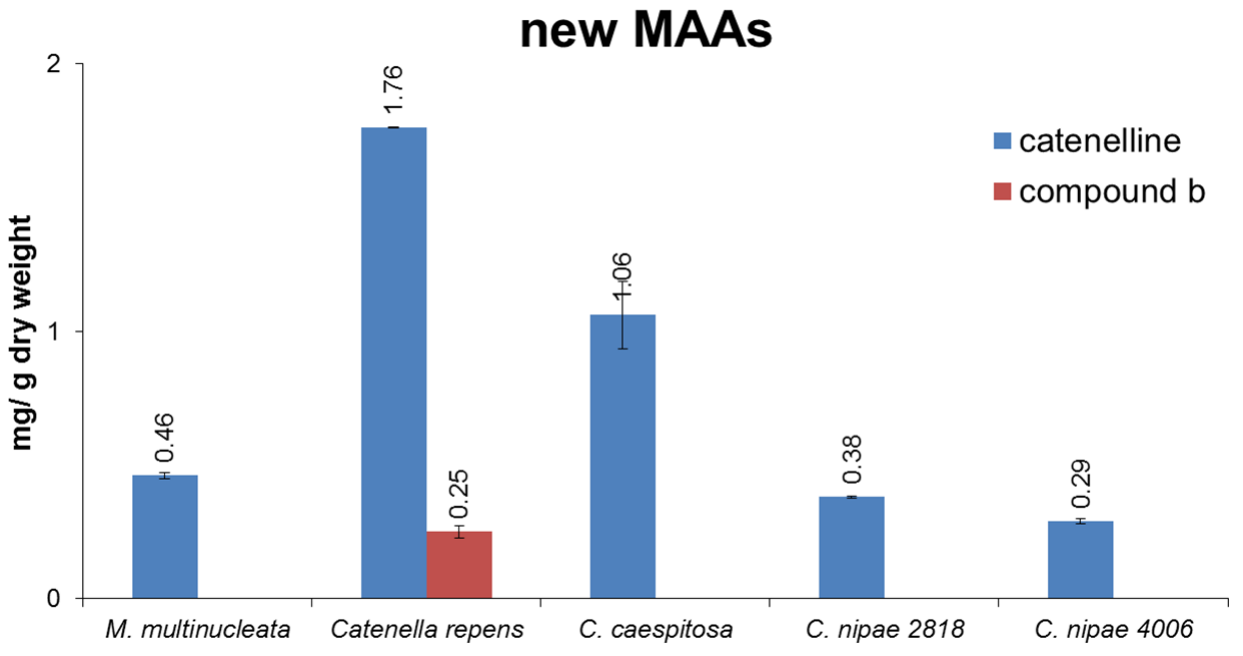
Summary of quantification results stated as mg MAA/g dry weight. In the first diagram, the results of known MAAs are presented (*n* = 3); the second diagram illustrates the content of catenelline and compound **b**, a not yet identified MAA (*n* = 3).

As can be seen from typical chromatograms shown in Figure 5, the developed assay was well suited to analyze algal extracts. For example, in *Palmaria palmata* four of the five standards were clearly assignable based on matching retention times, characteristic UV-spectra and LC-MS data. The latter are shown as extracted ion chromatograms (EIC), where only ions typical for each particular MAA are depicted. In addition to porphyra-334, shinorine, palythine and asterina-330 an *m*/*z* value typical for palythinol was found in this sample; thus, this signal was tentatively assigned as such. Following the same strategy all analyzed samples were screened for the presence of MAAs, and then respective signals were quantified based on peak area; Figure 6 shows the results in mg MAA per g of dried alga. No MAAs were found in *Leptolyngbya foveolarum* and in *Calothrix* sp.; yet, the chromatograms revealed signals with absorption maxima typical for MAAs. Their molecular weight, however, was much higher than known derivatives, so that glycosylated MAAs, as already described for *Nostoc commune*, might be present [17]. In all investigated *Catenella* samples catenelline was the dominant MAA, with a content ranging from 0.29 (*C. nipae* 4006) to 1.76 mg/g dry weight (*C. repens*). Rather surprisingly, the terrestrial green alga *Macrochloris multinucleata* also showed a signal with matching retention time and molecular mass; its calculated content was 0.46 mg/g dry weight. However, despite this evidence, for an unambiguous assignment as catenelline further isolation and elucidation of the respective compound will be required. All other analyzed samples contained either palythine (*Palmaria palmata*, 9.94 mg/g dry weight; *Nostoc commune*, 0.57 mg/g dry weight) or porphyra-334 (both *Porphyra* species; 6.82 to 10.85 mg/g dry weight) as dominant MAAs. *Lichina pygmea* was the only species containing detectable amounts of mycosporine-serinol (1.90 mg/g dry weight).

## 3. Discussion

Mycosporine-like amino acids, which are the most common UV absorbing secondary metabolites in many algae and cyanobacteria, have been studied extensively in the past. However, there is still a demand for improved analytical procedures for their qualitative and quantitative determination. First, mostly due to a lack of standards, they were often quantified using “only” their specific extinction coefficients (ε); second, their isolation is tricky and extremely high ε values might lead to false “pure compounds”, thus purity has to be confirmed by NMR; third, their polar nature renders an analysis on conventional RP material difficult; and fourth, none of the published assays were fully validated. All of these facts indicate that, with the currently available techniques and protocols, the quantitative analysis of MAAs was always associated with a certain degree of uncertainty.

The HILIC method presented here minimizes all of the aforementioned problems. This rather new type of stationary phase was well suited for the fast and selective separation of five MAAs, whose identity and purity was confirmed by LC-MS and NMR. In contrast to RP based methods, the compounds did not elute close to the void volume but with adequate retention times. After a careful method optimization, the assay was validated, and three of the standards (palythine, shinorine and porphyra-334), which were available in sufficient amounts, utilized for calibration. As a volatile mobile phase was used the direct coupling to MS was possible, whereby HILIC offers a kinetic advantage due to higher solute diffusivity and therefore an increased sensitivity in ESI-MS [18]. Respective experiments indicated the presence of unknown MAAs in *Catenella* species. The dominant of the two compounds could be isolated by chromatographic means and its structure elucidated based on LC-MS and NMR data. It turned out to be a novel amino-cyclohexenimine MAA with a serine and taurine residue; “catenelline” is proposed as its name. It is known that taurine can be produced in high amounts in red algae, but it is not an essential organic acid [19]. In higher plants taurine acts as an anti-stress agent [20], and in algae it can serve as an osmolyte to tolerate high salt concentrations [21]. Thus, even if the primarily function of catenelline is that of an UV-protectant, other possible physiological functions are worth investigating further.

Members of the genus *Catenella* exhibit a rather specific ecology, since they grow as typical epiphytes on the pneumatophores, prop roots and basal trunks of tropical mangrove trees [16,22], as well as in temperate salt marshes or on exposed rocky shores. Consequently, these marine red algae occur under almost “terrestrial” conditions always in the upper littoral to supralittoral zone of the different habitats [16]. One feature of this high vertical position in the littoral zone is the regular exposure to tidal immersion-emersion cycles, resulting in strong diurnal and seasonal gradients of various physico-chemical parameters, such as salinity, temperature, radiation conditions and nutrient concentrations. Therefore, *Catenella* species have to cope with and acclimatize to these stressors, and in previous studies it could be shown that, for example, salinity fluctuations are compensated by the biosynthesis and accumulation of heteroside floridoside [23]. In addition, the same authors also reported the presence of a new metabolite isethionic acid, as well as an increase in certain signals (including “MAAs”) with rising salinities, but a proper quantification was not possible at that time due to a lack of standards. The present study fully confirms the first report on the occurrence of MAAs in *Catenella* species [16], and points to a general occurrence of catenelline among members of the genus *Catenella*.

MAAs are considered passive UV-sunscreens because they dissipate the absorbed short wavelength radiation energy in the harmless form of heat without generating photochemical reactions [24]. Above all, these biomolecules exhibit extremely high molar absorptivity for UV-A and UV-B and have been reported to be photochemically stable structures, both of which are prerequisites for their sunscreen function [25]. Besides their role as UV-sunscreen, some MAAs such as mycosporine-glycine also show moderate antioxidant activity [26]. The photo-physicochemical properties of MAAs guarantee a high UV-protective effectiveness in combination with antioxidant capabilities, which seems to be particularly important in “terrestrial” habitats such as the upper littoral to supralittoral zone of tropical mangroves or temperate rocky shores. Their increased production will depend on environmental conditions like the degree of UV irradiation. Accordingly, our quantitative results will possibly not reflect the real life scenario. In this study, the organisms were grown in the lab under low light conditions, and their analysis should indicate the practical applicability of the developed HILIC method “only”. Field collected samples could therefore show much higher MAA levels, also including those species where no respective compounds were found.

## 4. Experimental

### 4.1. Reagents and Chemicals

All solvents used for isolation and analytical studies were of analytical grade and purchased from Merck (Darmstadt, Germany). Inorganic salts and vitamins for the culture media, as well as ammonium acetate, were purchased from Sigma Aldrich (Vienna, Austria). Soil for preparing the algal culture media was commercially available material (Floragard flower soil, Oldenburg, Germany). HPLC grade water was produced by a Satorius arium 611 UV water purification system (Göttingen, Germany).

### 4.2. Biological Material and Cultivation Methods

The investigated algae and their origin are summarized in Table 3; the samples were identified by one of the authors (U.K.) or obtained as pure-culture strains. The latter (*Macrochloris multinucleata*, *Calothrix* sp., *Leptolyngbya foveolarum* and *Nostoc commune*) were cultivated in Erlenmeyer flasks, either using Bold’s Basal medium (for green algae) or BG-11 media (for cyanobacteria). The flasks were kept under controlled growth conditions at 20 °C and continuous light (35 µM photons m^−2^·s^−1^ PAR), following a light-dark rhythm of 16 to 8 h. In the exponential growth phase each culture was transferred into fresh media. *Catenella repens* was collected in the field, rinsed with sea-water, air-dried in the sun for 30–45 min and stored directly afterwards in sealed plastic bags under cool, dry and dark conditions. The other *Catenella* samples were grown as unialgal cultures in the lab. They were isolated from field collections, established as laboratory culture and maintained in marine media with a salinity of 30 enriched with PES/2 [27] at 25 °C; light was supplied with 30 µM photons m^−2^·s^−1^ PAR, cool-white fluorescent lighting (Osram daylight, Munich, Germany), and in a 16 h light to 8 h dark photoperiod. The algae were maintained in continuous immersion culture with changes of medium at intervals of 4 weeks. After sufficient biomass was grown, samples were oven-dried at 45 °C overnight and sent to Innsbruck for MAA analysis. To ensure stability of selected samples they were repeatedly assayed for their MAA content, *i.e.*, *C. repens* was analyzed at least once per year over a period of 9 years during routine measurements of various algal samples and no degradation in MAA content was detected. The same was true for ocular lenses of the coral trout, which contains asterina-330.

### 4.3. Isolation and Structural Analysis of MAAs

Catenelline was isolated from the epilithic red alga *Catenella repens*. The dried material (45 g) was extracted as described under 4.4, only solvent ratios were adapted. The dried methanolic extract (1.2 g) was re-dissolved in water and applied on activated carbon SPE cartridges (Supelclean Envi-Carb, size: 12 mL with 1 g material) from Supelco (Vienna, Austria). After washing with water, the MAA enriched fraction (110 mg) was eluted with methanol. A final purification of catenelline was achieved by semi-preparative HPLC (Dionex UltiMate 3000, Thermo, Waltham, MA, USA), using a Luna HILIC column (250 mm × 4.6 mm, 5 µm; Phenomenex, Aschaffenburg, Germany) as stationary phase. The mobile phase comprised acetonitrile/water (9:1) with 5 mM ammonium acetate (A) and acetonitrile/water (1:1) with 5 mM ammonium acetate (B), applied in gradient mode (100% A in 25 min to 30% A/70% B). The separation was monitored at 320 nm, column temperature and flow rate were set to 30 °C and 1.0 mL/min, respectively. Per run, 50 µL were injected (25 mg/mL sample concentration), and after approximately 40 repetitions, 3 mg of pure catenelline was obtained.

Standards of known MAAs were isolated from two commercially available algae (*Palmaria palmata* and *Porphyra* sp.), with the exception of mycosporine-serinol (**2**; isolated from *Lichina pygmea*), following a previously reported protocol [14]. Asterina-330 (**5**), isolated from the ocular lenses of *Plectropomus leopardus*, was supplied by U.K. 1D- and 2D-NMR data for all isolated compounds were recorded at 25 °C on an Ultra-Shield 600 MHz instrument (Bruker, Bremen, Germany). The samples were dissolved in deuterated water and tetramethylsilan was used as internal standard (Euriso-Top, Saint-Aubin, France). NMR data of the known MAAs (see Table S1, Supplementary Materials) were in good agreement with literature [28,29].

### 4.4. Sample Preparation

The powdered dried algae (0.2 g) was soaked overnight in 1 mL water at 4 °C, and extracted four times by 30 min of sonication with 10 mL methanol each (Sonorex 35 KHz, Bandelin, Berlin, Germany) [11]. After centrifugation (1000× *g* for 5 min), the supernatant was combined and evaporated to dryness at 40 °C in a rotary evaporator (Büchi, Flawil, Switzerland). For final HPLC and LC-MS analysis solutions with a concentration of 1–10 mg/mL were prepared in water, depending on the MAA concentration.

### 4.5. Analytical Conditions

Experiments were performed on an Agilent 1200 HPLC (Waldbronn, Germany), using a HILIC Poroshell 120 column (150 mm × 4.6 mm, 2.7 µm) from Agilent. The mobile phase comprised acetonitrile/water (9:1) with 5 mM ammonium acetate (A) and acetonitrile/water (1:1) with 5 mM ammonium acetate (B). Elution in gradient mode (60% A/40% B to 100% B in 30 min) was performed, followed by 15 min of re-equilibration with 60% A. The DAD was set to 320 nm, and flow rate, sample volume and column temperature were adjusted to 0.3 mL/min, 10 µL and 20 °C, respectively. HPLC-MS experiments were carried out on an Agilent 1260 HPLC system coupled to an amaZon iontrap mass spectrometer (Bruker, Bremen, Germany). The chromatographic conditions were as described before, MS-spectra were recorded in positive ESI mode, with a drying gas temperature of 200 °C, the nebulizer gas (nitrogen) set to 23 psi, and a nebulizer flow (nitrogen) of 8 L/min. The scanned mass range was between *m*/*z* 100 and 1500, at a capillary voltage of 4.5 kV. Additionally, the exact mass of the novel compound catenelline was determined in positive ESI mode on a micrOTOF-Q II MS (Bruker, Bremen, Germany). Here the settings were: nebulizer gas: 23.2 psi, dry gas: 8 L/min, dry temperature: 220 °C, capillary voltage: 3.0 kV, collision energy: 10.0 eV, and a transfer time of 90.0 µs.

### 4.6. Method Validation

In order to establish calibration curves, a single stock solution with **1** (1.0 mg/mL), **3** (0.5 mg/mL) and **4** (1.0 mg/mL) in water was prepared. Individual calibration levels were obtained by serial dilution, and each solution was analyzed under optimum HPLC conditions in triplicate. Limit of detection and limit of quantification were evaluated by visually defining concentrations equivalent to S/N ratios of 3 (LOD) and 10 (LOQ). Accuracy was determined by spiking three aliquots of the sample *Leptolyngbya foveolarum* with three different concentrations of the standards, followed by extraction and analysis. The method’s precision was confirmed by its repeatability, as well as inter- and intra-day variation, which were determined for palythine in *Palmaria palmata*. For this purpose, five individual samples were extracted and analyzed on three consecutive days (Table 1).

## Supplementary Files

Supplementary File 1

## References

[1] Körner C. (2003). Climatic Stress. Alpine Plant Life.

[2] Larcher W.C., Kainmüller C., Wagner J. (2010). Survival types of high mountain plants under extreme temperatures. Flora.

[3] Rastogi R.P., Incharoensakdi A. (2014). Characterization of UV-screening compounds, mycosporine-like amino acids, and scytonemin in the cyanobacterium *Lyngbya* sp. CU2555. FEMS Microbiol. Ecol..

[4] Rastogi R.P., Sonani R.R., Madamwar D. (2014). The high-energy radiation protectant extracellular sheath pigment scytonemin and its reduced counterpart in the cyanobacterium *Scytonema* sp. R77DM. Bioresour. Technol..

[5] Karsten U. (2008). Defence strategies of algae and cyanobacteria against solar ultraviolet radiation. Algal Chemical Ecology.

[6] Flaim G., Obertegger U., Anesi A., Guella G. (2014). Temperature-induced changes in lipid biomarkers and mycosporine-like amino acids in the psychrophilic dinoflagellate *Peridinium aciculiferum*. Freshw. Biol..

[7] Karsten U., Bischof K., Hanelt D., Tüg H., Wiencke C. (1999). The effect of ultraviolet radiation on photosynthesis and ultraviolet-absorbing substances in the endemic Arctic macroalga *Devaleraea ramentacea* (Rhodophyta). Physiol. Plant..

[8] Takano S., Uemura D., Hirata Y. (1978). Isolation and structure of 2 new amino-acids, palythinol and palythene, from zooanthid *Palythoa tuberculosa*. Tetrahedron Lett..

[9] Carreto J.I., Carignan M.O. (2011). Mycosporine-like amino acids: Relevant secondary metabolites. Chemical and ecological aspects. Mar. Drugs.

[10] Whitehead K., Hedges J.I. (2002). Analysis of mycosporine-like amino acids in plankton by liquid chromatography electrospray ionization mass spectrometry. Mar. Chem..

[11] Carreto J.I., Carignan M.O., Montoya N.G. (2005). A high-resolution reverse-phase liquid chromatography method for the analysis of mycosporine-like amino acids (MAAs) in marine organisms. Mar. Biol..

[12] Stochaj W.R., Dunlap W.C., Shick J.M. (1994). Two new UV-absorbing mycosporine-like amino-acids from the sea-anemone *Anthopleura elegantissima* and the effects of zooxanthellae and spectral irradiance on chemical composition and content. Mar. Biol..

[13] Volkmann M., Gorbushina A.A. (2006). A broadly applicable method for extraction and characterization of mycosporines and mycosporine-like amino acids of terrestrial, marine and freshwater origin. FEMS Microbiol. Lett..

[14] Shick J.M., Dunlap W.C., Pearse J.S., Pearse V.B. (2002). Mycosporine-like amino acid content in four species of sea anemones in the genus *Anthopleura* reflects phylogenetic but not environmental or symbiotic relationships. Biol. Bull..

[15] Hartmann A., Gostner J., Fuchs J.E., Chaita E., Aligiannis N., Skaltsounis L., Ganzera M. (2015). Inhibition of collagenase by mycosporine-like amino acids from marine sources. Planta Med..

[16] Karsten U., Sawall T., West J., Wiencke C. (2000). Ultraviolet sunscreen compounds in epiphytic red algae from mangroves. Hydrobiologia.

[17] Matsui K., Nazifi E., Kunita S., Wada N., Matsugo S., Sakamoto T. (2011). Novel glycosylated mycosporine-like amino acids with radical scavenging activity from the cyanobacterium *Nostoc commune*. J. Photochem. Photobiol. B.

[18] Heaton J., Smith N.W. (2012). Advantages and disadvantages of HILIC: A brief overview. Chromatogr. Today.

[19] McCusker S., Buff P.R., Yu Z., Fascetti J.A. (2014). Amino acid content of selected plant, algae and insect species: A search for alternative protein sources for use in pet foods. J. Nutr. Sci..

[20] Lee D.H. (2015). *In vitro* analysis of taurine as anti-stress agent in tomato (*Solanum lycopersicum*)-Preliminary study. Adv. Exp. Med. Biol..

[21] Tevatia R., Allen C., Rudrappa D., White D., Clemente T.E., Cerutti H., Demirel Y., Blum P. (2015). The taurine biosynthetic pathway of microalgae. Algal Res..

[22] Dawes C.J. (1996). Macroalgal diversity, standing stock and productivity in a northern mangalon the west coast of Florida. Nova Hedwig..

[23] Karsten U., Barrow K.D., Mostaert A.S., King R.J. (1995). The osmotic significance of the heteroside floridoside in the mangrove alga *Catenella nipae* (Rhodophyta: Gigartinales) in Eastern Australia. Estuar. Coast. Shelf Sci..

[24] Bandaranayake W.M. (1998). Mycosporines: Are they nature’s sunscreens?. Nat. Prod. Rep..

[25] Conde F.R., Churio M.S., Previtali C.M. (2000). The photoprotector mechanism of mycosporine-like amino acids. Excited-state properties and photostability of porphyra-334 in aqueous solution. J. Photochem. Photobiol. B.

[26] Dunlap W.C., Yamamoto Y. (1995). Small-molecule antioxidants in marine organisms: Antioxidant activity of mycosporine-glycine. Comp. Biochem. Physiol. B.

[27] Starr R.C., Zeikus J.A. (1993). UTEX: The culture collection of algae at the University of Texas at Austin, 1993 list of cultures. J. Phycol..

[28] La Barre J.M.K., Kornprobst J.M. (2014). Outstanding Marine Molecules.

[29] Favre-Bonvin J., Arpin N., Brevard C. (1976). Structure of mycosporine (P-310). Can. J. Chem..

