# Supplementary material for: Analysis of Mycosporine-Like Amino Acids in Selected Algae and Cyanobacteria by Hydrophilic Interaction Liquid Chromatography and a Novel MAA from the Red Alga Catenella repens
Source: Mar Drugs. 2015 Oct 9;13(10):6291–305. doi: 10.3390/md13106291 (PMC4626690; doi:10.3390/md13106291)
Supplement: Supplementary File 1 [file marinedrugs-13-06291-s001.docx]

**Supplementary Materials**

Influence of temperature on the HILIC separation, ^1^H- and ^13^C-NMR shift values of porphyra-334, shinorine and palythine, as well as the NMR spectra (^1^H, ^13^C, COSY, HMBC and HSQC) for catenelline and its MS/MS fragmentation pattern are shown as supplementary material.


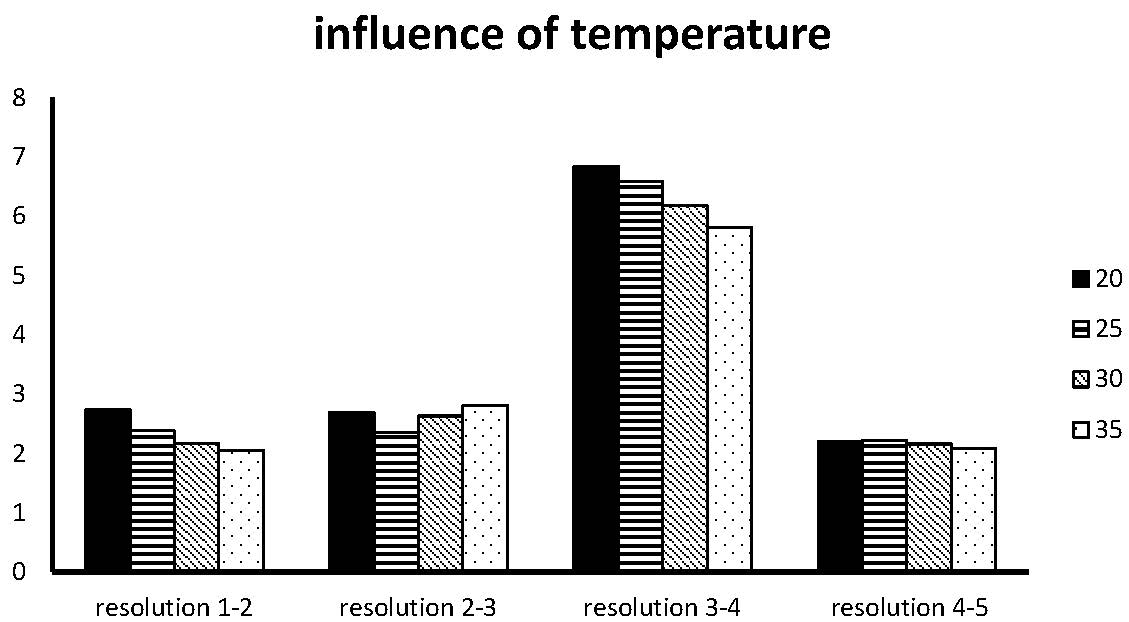


**Figure S1.** Influence of temperature on the separation (resolution) of compounds **1**–**5**.


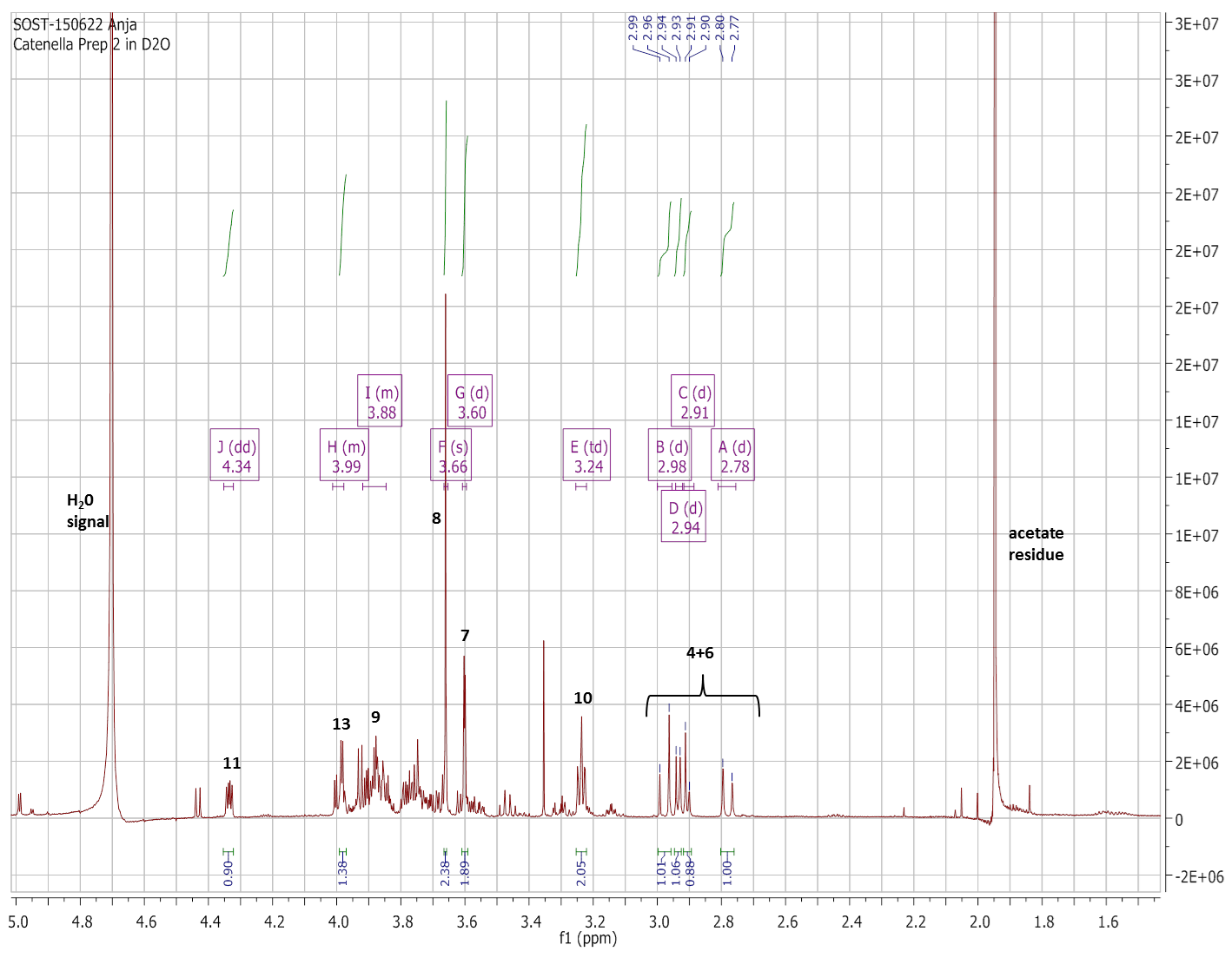


**Figure S2.** ^1^H-NMR spectra of the novel MAA catenelline, recorded in deuterated water on a 600 MHz NMR instrument.


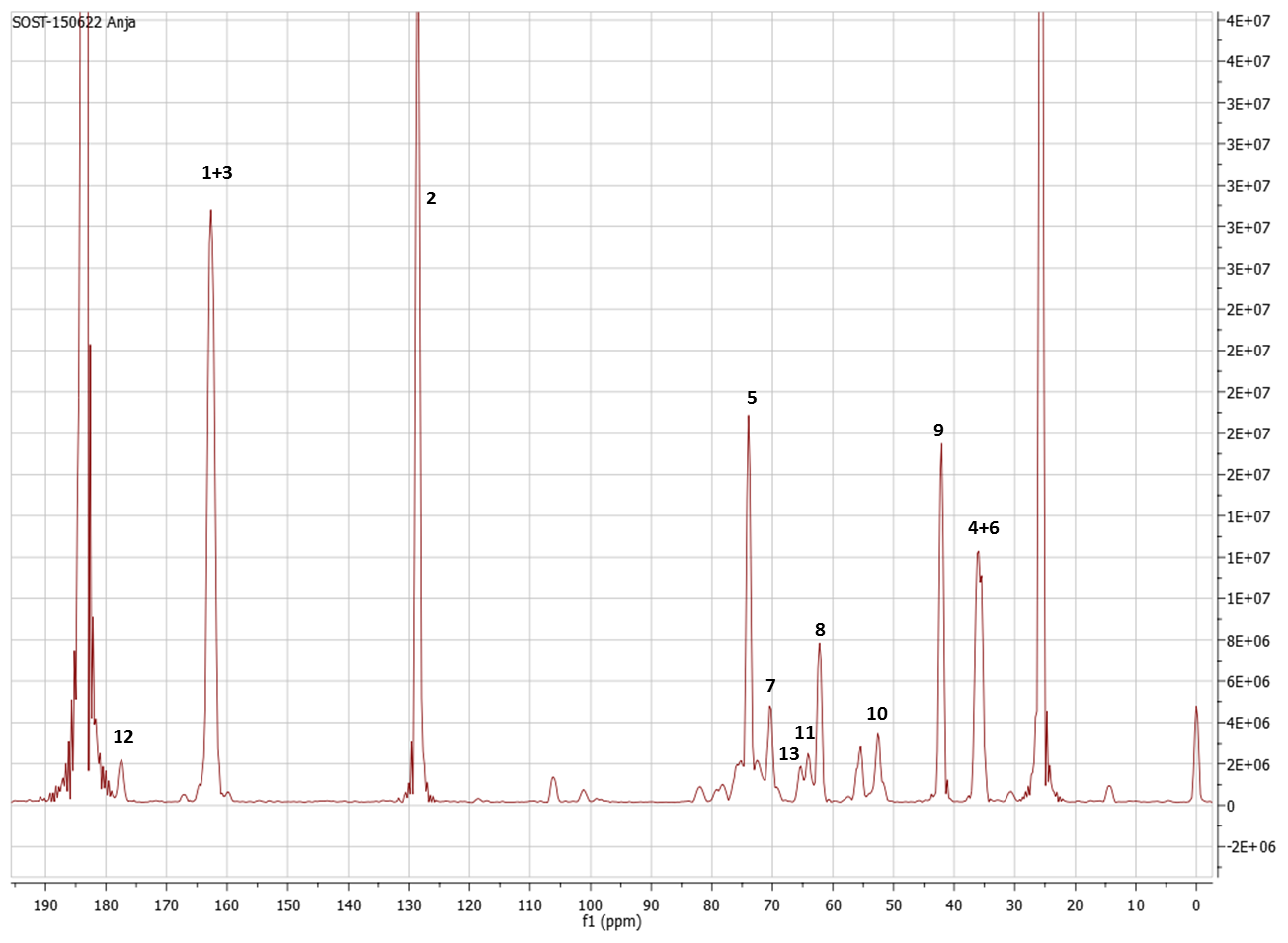


**Figure S3.** ^13^C-NMR spectra of the novel MAA catenelline, recorded in deuterated water on a 600 MHz NMR instrument.


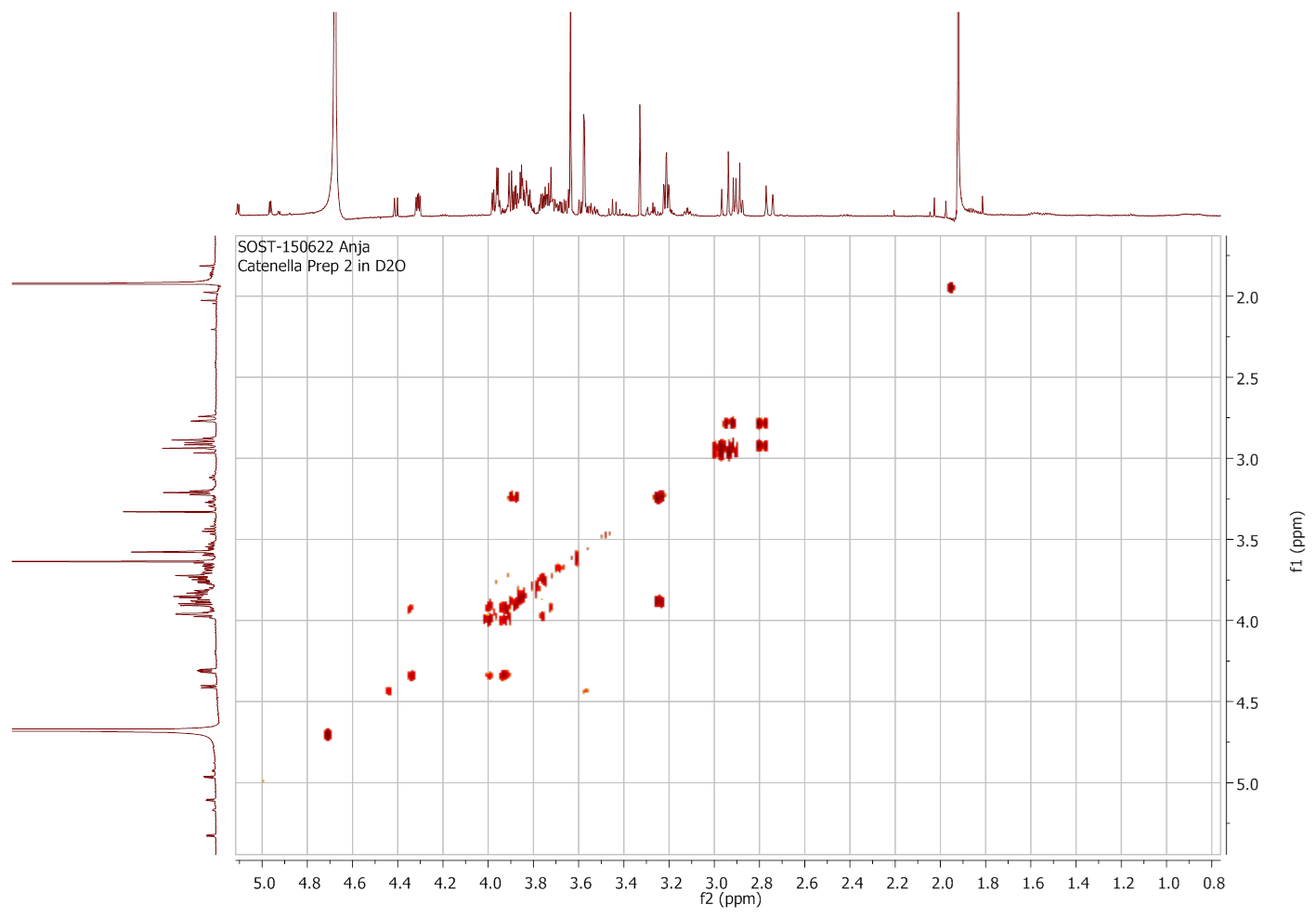


**Figure S4.** COSY-spectra of the novel MAA catenelline, recorded in deuterated water on a 600 MHz NMR instrument.


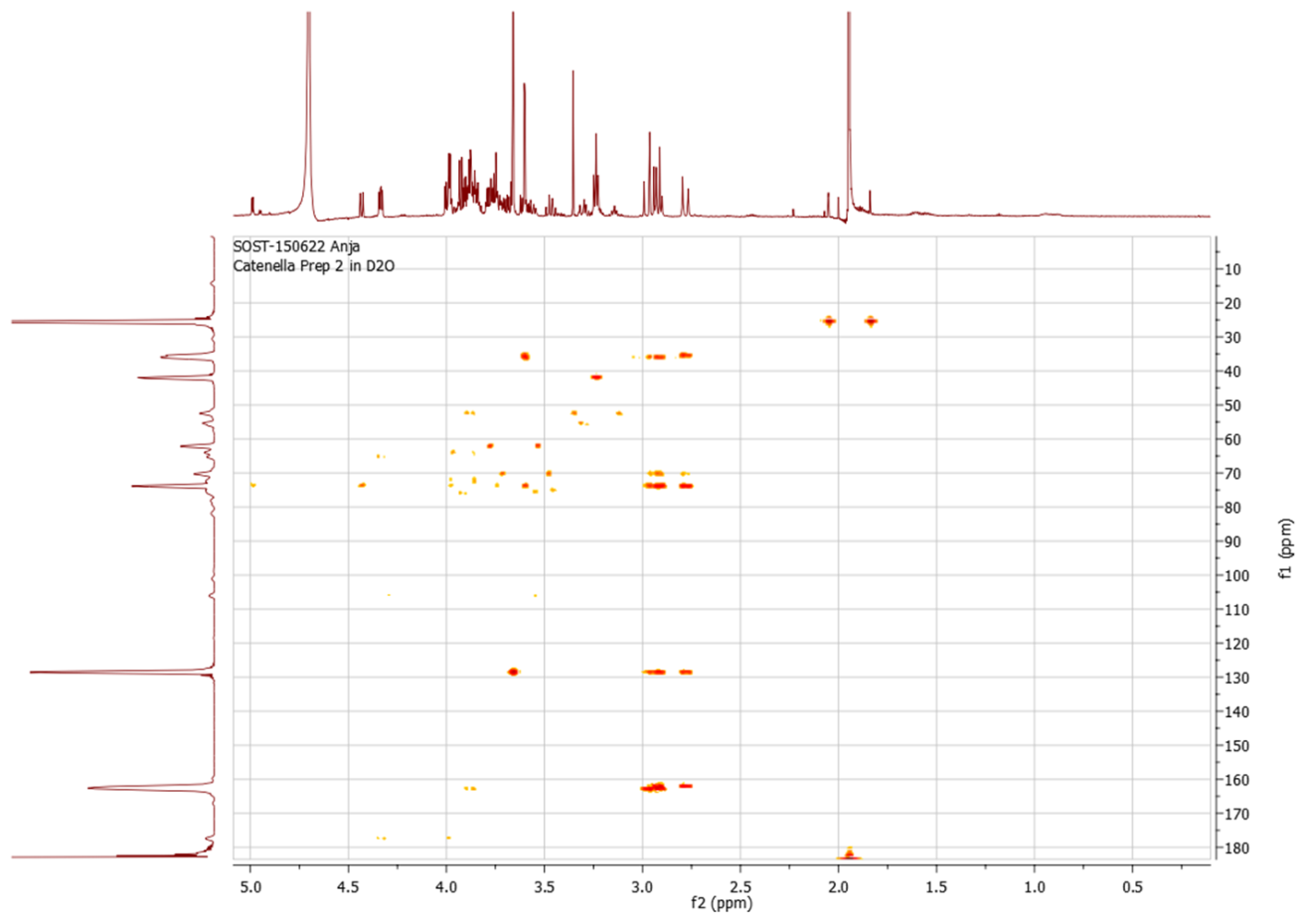


**Figure S5.** HMBC spectra of the novel MAA catenelline, recorded in deuterated water on a 600 MHz NMR instrument.


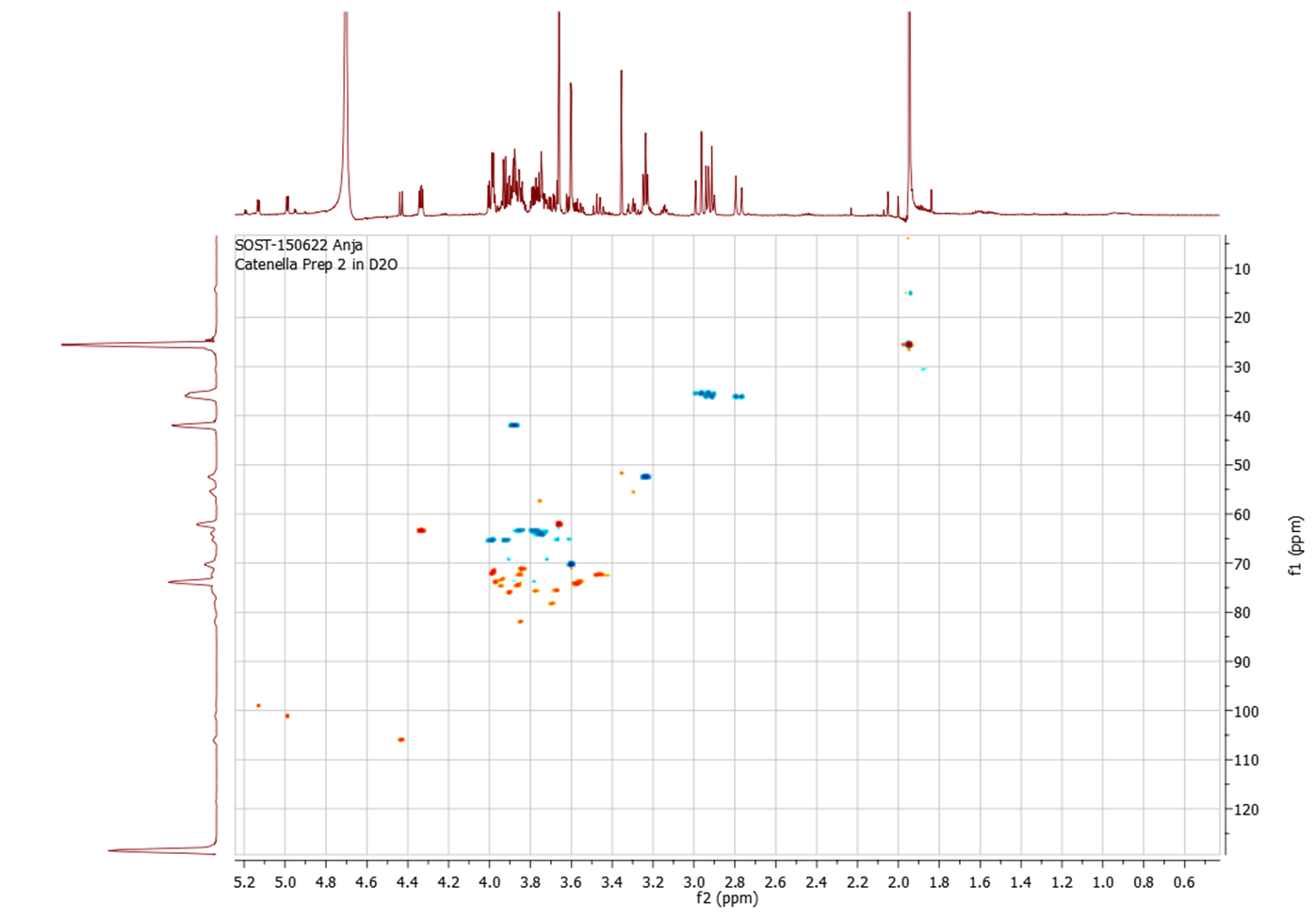


**Figure S6.** HSQC spectra of the novel MAA catenelline, recorded in deuterated water on a 600 MHz NMR instrument.


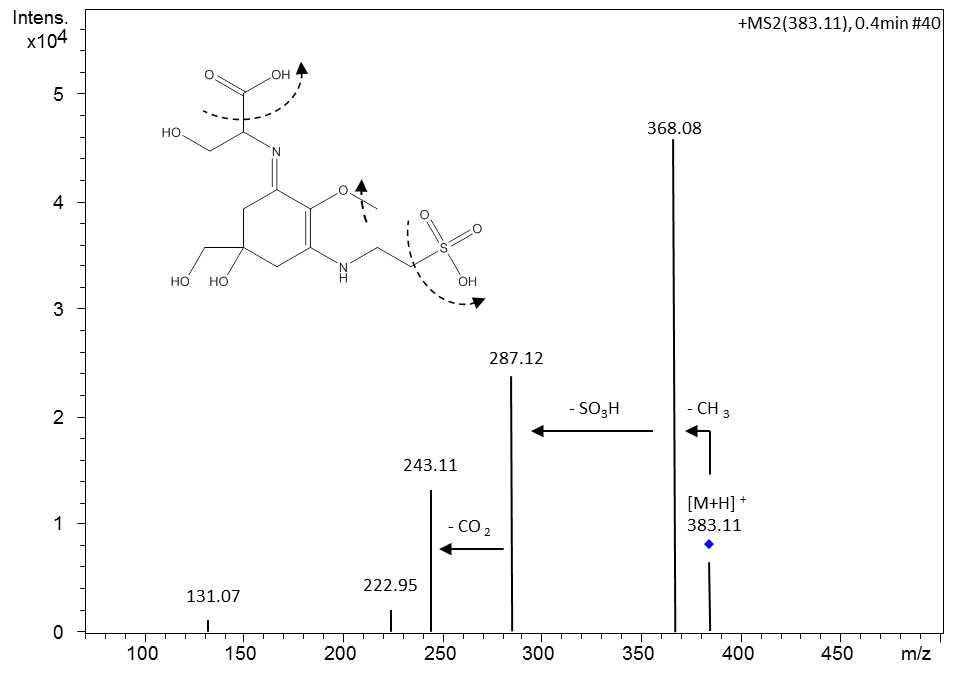


**Figure S7.** Fragmentation pattern of cantenelline, as obtained by direct infusion (5 µL/min) of an aqueous solution (concentration 0.2 mg/mL). MS-conditions: amaZon iontrap mass spectrometer, positive ESI mode, drying gas temperature: 200 °C, nebulizer gas (nitrogen): 10 psi, nebulizer flow (nitrogen): 3 L/min, capillary voltage: 4.5 kV, auto MS/MS mode.

**Table S1.** NMR shift values of the isolated MAAs porphyra, shinorine, palythine and mycosporine-serinol.

|  | **Porphyra** | | **Shinorine** | | **Palythine** | | **Mycosporine-Serinol** | |
| --- | --- | --- | --- | --- | --- | --- | --- | --- |
|  | ^13^C | ^1^H | ^13^C | ^1^H | ^13^C | ^1^H | ^13^C | ^1^H |
| 1 | 162.22 | - | 162.56 | - | 164.7 | - | 188.52 | - |
| 2 | 128.73 | - | 128.73 | - | 127.6 | - | 133.65 | - |
| 3 | 163.71 | - | 163.79 | - | 163.6 | - | 161.72 | - |
| 4 | 31.83 | 2.75 (m) | 35.49 | 2.78 (m) | 36.14 | 2.72 (m) | 45.71 | 2.68 (dd) |
| 5 | 73.03 | - | 73.83 | - | 74.03 | - | 64.91 | - |
| 6 | 35.84 | 2.85 (m) | 36.15 | 2.87 (m) | 38.59 | 2.90 (m) | 36.42 | 2.41 (dd) |
| 7 | 67.33 | 3.57 (s) | 68.58 | 3.56 (s) | 70.13 | 3.56 (s) | 61.77 | - |
| 8 | 56.62 | 3.69 (s) | 56.50 | 3.66 (s) | 61.71 | 3.63 (s) | 60.81 | - |
| 9 | 52.23 | 4.09 (s) | 58.96 | 4.09 (s) | 54.84 | 4.13 (s) | 73.45 | 3.67 (m) |
| 10 | 176.26 | - | 177.73 | - | 176.66 | - | 73.45 | 3.67 (m) |
| 11 | 62.57 | 4.12 (d) | 62.82 | 4.48 (m) |  |  | 61.77 | 3.7 (m) |
| 12 | 179.39 | - | 179.35 | - |  |  |  |  |
| 13 | 70.79 | 4.32 (m ) | 52.97 | 3.85 (dd ) |  |  |  |  |
| 14 | 20.17 | 1.25 (d ) |  |  |  |  |  |  |

All spectra were recorded in deuterated water, using TMS (tetramethylsilane) as internal standard, and measured on a 600 MHz NMR instrument.

© 2015 by the authors; licensee MDPI, Basel, Switzerland. This article is an open access article distributed under the terms and conditions of the Creative Commons Attribution license (http://creativecommons.org/licenses/by/4.0/).
